# Characterization of the Regulatory Mechanisms of Activating Transcription Factor 3 by Hypertrophic Stimuli in Rat Cardiomyocytes

**DOI:** 10.1371/journal.pone.0105168

**Published:** 2014-08-19

**Authors:** Elina Koivisto, Alicia Jurado Acosta, Anne-Mari Moilanen, Heikki Tokola, Jani Aro, Harri Pennanen, Hanna Säkkinen, Leena Kaikkonen, Heikki Ruskoaho, Jaana Rysä

**Affiliations:** 1 Department of Pharmacology and Toxicology, Institute of Biomedicine, University of Oulu, Oulu, Finland; 2 Department of Pathology, Institute of Diagnostics, University of Oulu, Oulu, Finland; 3 Division of Pharmacology and Pharmacotherapy, Faculty of Pharmacy, University of Helsinki, Helsinki, Finland; 4 School of Pharmacy, University of Eastern Finland, Kuopio, Finland; Max-Delbrück Center for Molecular Medicine (MDC), Germany

## Abstract

**Aims:**

Activating transcription factor 3 (ATF3) is a stress-activated immediate early gene suggested to have both detrimental and cardioprotective role in the heart. Here we studied the mechanisms of ATF3 activation by hypertrophic stimuli and ATF3 downstream targets in rat cardiomyocytes.

**Methods and Results:**

When neonatal rat cardiomyocytes were exposed to endothelin-1 (ET-1, 100 nM) and mechanical stretching in vitro, maximal increase in ATF3 expression occurred at 1 hour. Inhibition of extracellular signal-regulated kinase (ERK) by PD98059 decreased ET-1– and stretch–induced increase of ATF3 protein but not ATF3 mRNA levels, whereas protein kinase A (PKA) inhibitor H89 attenuated both ATF3 mRNA transcription and protein expression in response to ET-1 and stretch. To characterize further the regulatory mechanisms upstream of ATF3, p38 mitogen-activated protein kinase (MAPK) signaling was investigated using a gain-of-function approach. Adenoviral overexpression of p38α, but not p38β, increased ATF3 mRNA and protein levels as well as DNA binding activity. To investigate the role of ATF3 in hypertrophic process, we overexpressed ATF3 by adenovirus-mediated gene transfer. *In vitro*, ATF3 gene delivery attenuated the mRNA transcription of interleukin-6 (IL-6) and plasminogen activator inhibitor-1 (PAI-1), and enhanced nuclear factor-κB (NF-κB) and Nkx-2.5 DNA binding activities. Reduced PAI-1 expression was also detected *in vivo* in adult rat heart by direct intramyocardial adenovirus-mediated ATF3 gene delivery.

**Conclusions:**

These data demonstrate that ATF3 activation by ET-1 and mechanical stretch is partly mediated through ERK and cAMP-PKA pathways, whereas p38 MAPK pathway is involved in ATF3 activation exclusively through p38α isoform. ATF3 activation caused induction of modulators of the inflammatory response NF-κB and Nkx-2.5, as well as attenuation of pro-fibrotic and pro-inflammatory proteins IL-6 and PAI-1, suggesting cardioprotective role for ATF3 in the heart.

## Introduction

Most patients with heart failure have a history of left ventricular hypertrophy, which is initially an adaptive response to increased work load. However, after sustained external load, hearts can evolve to a state of decompensated hypertrophy resulting in the dilatation of the left ventricle and loss of contractile function [1], [2]. In response to hypertrophic stimuli, a fundamental reprogramming occurs within the adult cardiomyocytes that results in the expression of genes encoding fetal protein isoforms. The immediate early genetic response includes transcription of genes such as c-*fos*, c-*jun* and early growth response–1 (EGR-1). Later during the hypertrophic process, the transcription of contractile proteins, α-myosin heavy chain (α-MHC) and cardiac α-actin, are down-regulated and expression of β-MHC and skeletal muscle α-actin are up-regulated. Also non-contractile proteingenes such as atrial natriuretic peptide (ANP) and B-type natriuretic peptide (BNP) become highly expressed within ventricular myocytes [3].

The myocardium can hypertrophy in response to increases in wall stress as well as to humoral and neural stimuli [4]. Wall stretch and various other hypertrophic stimuli such as angiotensin II, endothelin-1 (ET-1), cytokines and growth factors in turn result in the activation of specific intracellular signalling cascades including mitogen-activated protein kinase (MAPK), protein kinase C (PKC), insulin-like growth factor-1/Akt, and the calcium-activated protein phosphatase calcineurin [5]. Specifically, all MAPK pathways – extracellular signal-regulated kinase (ERK), c-Jun N-terminal kinase (JNK) and p38 MAPK – are key signaling routes in mechanical load–induced hypertrophic process [6]. These and other intracellular signalling cascades then modulate transcription factors such as activator protein-1 (AP-1) [7], GATA-4, Nkx-2.5 and nuclear factor-κB (NF-κB) [8], which in turn regulate gene expression to facilitate the growth of the heart activated by mechanical load.

Our DNA microarray study using adenovirus-mediated overexpression of wild type (WT) p38α and constitutively active upstream MAP kinase kinase 3b (MKK3b) *in vivo* identified several novel p38 MAPK target genes, including activating transcription factor 3 (ATF3) [12]. ATF3 is a member of the ATF/cyclicAMP–responsive element-binding (ATF/CREB) family of transcription factors. In the heart, ATF3 transcription has been shown to be induced under oxidative stress (H_2_O_2_-treatment) as well as ischemia/hypoxia and ischemia-reperfusion models both *in vivo* and *in vitro* [9]–[11]. Recently, administration of pressor substances angiotensin II and phenylephrine (PE) in mice have been shown to up-regulate cardiac ATF3 [13]–[15]. However, the precise mechanisms regulating ATF3 activation during hypertrophic process are not fully understood, and whether mechanical stretch directly activates ATF3 in the heart is not known. Moreover, the exact role of ATF3 in the heart is not yet defined [15]–[19]. In a very recent study, cardiac overexpression of ATF3 in mice was sufficient to promote cardiac hypertrophy and exacerbate the deleterious effect of chronic pressure overload, while ATF3 knockout mice displayed less cardiac hypertrophy in the pressure overload model [18].

In the present study we demonstrate that ERK and PKA pathways are involved in ATF3 activation in response to ET-1 stimulation and mechanical stretching of neonatal rat cardiomyocytes (NRCM), and that the main p38 MAPK isoform upstream of ATF3 is p38α. We further show that ATF3 overexpression leads to a decreased rate of protein synthesis, a marker for cardiac hypertrophy, and is characterized with activation of a suppressor of inflammation, NF-κB, as well as with activation of a survival factor Nkx-2.5. In addition, ATF3 overexpression inhibited the expression of pro-inflammatory protein plasminogen activator inhibitor-1 (PAI-1) both *in vitro* and *in vivo*, as well as the expression of inflammatory cytokine interleukin-6 (IL-6) *in vitro*. Altogether, these data indicate that although induced by hypertrophic stimuli, the activation of ATF3 might be cardioprotective and contribute to the beneficial, adaptive cardiomyocyte hypertrophy.

## Materials and Methods

### Ethics statement

All experimental protocols were approved by the Animal Use and Care Committee of the University of Oulu and the Provincial Government of Western Finland Department of Social Affairs and Health (ESAVI/4365/04.10.03/2011).

### Materials

Cell culture reagents (bovine serum albumin, calcium chloride, Dulbecco's Modified Eagle Medium: Nutrient Mixture F-12 (DMEM/F-12), phosphate buffered saline (PBS), insulin-transferrin sodium-selenite media supplement, L-glutamine, penicillin-streptomycin, sodium puryvate, 3′,3′,5′-triiodo-L-thyronine), ET-1, PE, lipopolysaccharide (LPS), phorbol 12-myristate 13-acetate (PMA), PKA inhibitor H89, protease- and phosphatase- inhibitor cocktails (used in protein extraction) and protein extraction detergent IGEPAL CA-630, as well as all the oligonucleotides were from Sigma-Aldrich (St. Louis, MO, USA). ERK inhibitor PD98059 and p38 MAPK inhibitor SB203580 were from Tocris Bioscience (Bristol, UK). Antibodies against ATF3, NF-κB, Nkx-2.5 and AP-1 were from Santa Cruz Biotechnology Inc (Santa Cruz, CA, USA). ECL Plus Western Blotting Detection System reagents, First-Strand cDNA Synthesis Kit and leucine L-[4,5-3H] were from GE Healthcare (Waukesha, WI, USA). Bio-Rad Protein Assay was from Bio-Rad Laboratories (Hercules, CA, USA). Secondary antibodies horseradish peroxidase (HRP)-linked anti-rabbit IgG and anti-mouse IgG were purchased from Cell Signaling Technology (Danvers, MA, USA). Antibody for glyceraldehyde 3-phosphate dehydrogenase (GAPDH) as well as collagenase type II Worthington (used in cell culture) was from Millipore (Billerica, MA, USA). Heat-inactivated fetal bovine serum (FBS) for cell culture was from Invitrogen (Carlsbad, CA, USA). Recombinant human acidic fibroblast growth factor (aFGF) was from R&D systems (Minneapolis, MN, USA). Optitran BA-S 85 nitrocellulose membranes were from Schleicher & Schuell BioScience (Dassel, Germany). Rigid bottomed cell culture plates were from Greiner Bio-one (Monroe, NC, USA).

### Adenoviruses

Adenoviruses containing the coding regions of rat constitutively active MKK3b, constitutively active MKK6b, WT p38α, and replication-deficient adenovirus RAd*lacZ* were all driven by cytomegalovirus immediately early promoter. The MKK3b, MKK6b and WT p38β adenoviruses have been described previously [20]. The ATF3–overexpressing adenovirus (serotype 5) was generated as previously described [21]. Briefly, a full-length coding region of ATF3 cDNA was subcloned into the SalI and HindIII sites of the pShuttle-CMV vector (Qbiogene Inc, Montreal, Canada). The sequences for the cloning primers used were as follows; ATF3 forward 5′- GCG TCG ACT GGA GCA AAA TGA TGC TTC AAC-3′ and reverse 5′- CCC AAG CTT TTA GCT CTG CAA TGT TCC TTC-3′. The pShuttle-CMV-LacZ was a commercial plasmid (Stratagene, La Jolla, CA, USA). Adenoviruses were prepared by standard protocols (QbiogeneInc, Montreal, Canada) and purified by centrifugation on iodixanol (OptiPrep, Axis-Shield PoC AS, Oslo, Norway). The adenoviral titers (infectious unit, ifu) were determined by AdEasy Viral Titer Kit (Stratagene, La Jolla, CA, USA). Infectious unit is biologically equivalent to plaque forming unit (pfu).

### Animals

Newborn, 2- to 4-day-old Sprague-Dawley (SD)- rats of both sexes and male 2- to 3-month-old Sprague-Dawley (SD) rats weighing from 250 to 300 g and from the colony of the Centre of Experimental Animals at the University of Oulu were used.

### Cell culture and transfection

Cell cultures of cardiac ventricular cells were prepared from 2-to-4-day-old Sprague-Dawley -rats using the collagenase dissociation method [22]. For stretch experiments, the cells were plated at Collagen type I –coated Bioflex 6-well plates (Flexcell International Corporation) at a density of 1.6×10^5^/cm^2^ in serum-containing medium overnight. Thereafter, NRCM were incubated in complete serum-free medium (CSFM). For experiments with adenoviruses, these were added to the CSFM 18–24 h after plating at 4 MOI (multiplicity of infection). The experiments were completed 24 hours after the transduction. When appropriate, ERK inhibitor PD98059, p38 MAPK inhibitor SB203580, or PKA inhibitor H89 were added to culture medium. Two hours after the insertion of kinase inhibitors, PE or ET-1 was added to the culture medium, alternatively the cells were subjected to mechanical stretch. When appropriate, NRCM were treated with LPS, PE, PMA or aFGF.

### Application of mechanical stretch

The cells were exposed to cyclic mechanical stretch for 1, 4, 12, 24 or 48 hours by applying a computer controlled (Flexercell Strain Unit FX-3000, Flexcell International Corporation) vacuum suction under the flexible-bottomed collagen I-coated 6-well cell culture plates, as previously described [22]. After experiments the cells were quickly frozen with nitrogen oxide at −70°C.

### Cardiac gene transfer in vivo

Adenovirus-mediated intramyocardial gene transfer of ATF3 and LacZ at 1×10^9^ pfu into the left ventricle (LV) free wall of male 2-month-old Sprague-Dawley –rats [12]. Three days following the gene transfer, the animals were sacrificed and LV tissue samples were stored at −70°C for later analysis. Animals were from the colony of the Centre for Experimental Animals at the University of Oulu.

### Total protein extraction and Western blotting

Total protein extraction and Western blot analyses were performed as previously described [22], [23] with the exception of single protease/phosphatase inhibitors being changed to protease-inhibitor cocktail (1∶100 volume), phosphatase-inhibitor cocktail (1∶100 volume) and 1 mM dithiothreitol (DTT) (1∶1000 volume). Proteins were detected by enhanced chemiluminescence reagents (ECL Plus Western Blotting Detection System) with Fujifilm LAS-3000 Imager (Fujifilm, Tokyo, Japan). The bands were quantified with Quantity One software (Bio-Rad Hercules, CA, USA).

### Nuclear protein extraction and EMSA

EMSA assay is used to study DNA-protein binding interactions and qualitatively to identify sequence-specific DNA-binding proteins. To extract the nuclear and cytosolic proteins from NRCM cultures, the cells were incubated in low salt buffer consisting of 10 mM HEPES (4-(2-hydroxyethyl)-1-piperazineethanesulfonic acid), 10 mMKCl, 0.1 mM EDTA and 0.1 mM EGTA supplemented with protease-, phosphatase-inhibitor cocktail (1∶100 volume), and 1 mM DTT (1∶1000 volume). Then, membrane proteins were solubilized and isolated by adding 10% IGEPAL CA-630 detergent. After centrifugation, the pellets containing the nuclei were resuspended in high salt buffer containing 20 mM HEPES, 0.4 M NaCl, 1 mM EDTA and 1 mM EGTA supplemented like the low salt buffer (see above). The entire procedure was carried out at 4°C. Protein concentration of each sample was determined with Bio-Rad Protein Assay (Bio-Rad Hercules, CA, USA). The double-stranded synthetic oligonucleotides for electrophoretic mobility shift assay (EMSA) containing binding sequences for NF-κB or AP-1 at BNP promoter, Nkx-2.5 at ANP promoter and ATF3 binding site at macrophage inflammatory protein −1β promoter were labelled with [α-^32^P]-dCTP as described earlier [12]. The sequences used for the EMSA probes are provided in *Table 1*. ATF3 binding activity was determined with 24-bp double-stranded DNA oligonucleotide probes containing the ATF3 binding site, previously shown to be a consensus binding site 5′-TGACGT^A^_C_^A^_G_-3′ [24], [25]. To confirm DNA sequence specificity of the protein DNA complex formation, competition experiments with 1-, 10-, and 100-molar excesses of non-radiolabeled (“cold”) ATF3 oligonucleotides with intact or mutated binding sites were performed. For competition and supershift experiments appropriate oligodeoxynucleotides or antibodies were added to reaction mixture 20 min before addition of labeled probe. After electrophoresis the gels were dried and exposed to PhosphorImager screens (Molecular Dynamics, Sunnyvale, CA), which were then scanned using Bio-Rad Molecular Imager FX Pro Plus (Bio-Rad Laboratories). All the results were quantified using the Quantity One software.

**Table 1:** Oligonucleotides for EMSA.

Probe	Sequence
AP-1	5′- GGAAGTGTTTTTGATGAGTCACCCCA -3′
ATF3	5′-CTCGATGCCATGACATCATCTTTA-3′
NF-κB	5′- AGTTGAGGGGACTTTCCCAGGCCA -3′
Nkx-2.5	5′- AGAGACCTTTGAAGTGGGGGCCTCTTGAGGCCCCG-3′

### Isolation and analysis of RNA

Total RNA from left ventricular tissue was isolated by the guanidine thiocyanate-CsCl method and from cultured cardiomyocytes with TRIzol Reagent according to the manufacturer's protocol (Invitrogen) by using Phase Lock Gel system (Eppendorf AG, Hamburg, Germany) [21]. Rat ATF3, BNP, ANP, IL-6, PAI-1, osteopontin (OSP), bone morphogenetic protein-2 (BMP-2) and ribosomal 18S mRNA levels were measured by real-time RT-qPCR as previously described [12]. The primers and fluorogenic probes used are listed in *Table 2*. The results were normalized to 18S quantified from the same samples and then to control values.

**Table 2:** Sequences of rat forward (F) and reverse (R) primers and fluorogenic probes used for real time RT-qPCR analysis (sequences 5′ to 3′).

Gene	Primer	Fluorogenic probe
ANP	(F) GAAAAGCAAACTGAGGGCTCTG	TCGCTGGCCCTCGGAGCCT
	(R) CCTACCCCCGAAGCAGCT	
ATF-3	(F) TGAAGAATGAGAAGCAGCATCTG	TGCTCAACCTGCACCGGCCC
	(R) TCTGAGCCCGGAC GATACAC	
BMP-2	(F) ACACCGTGCTCAGCTTCCAT	ACGAAGAAGCCATCGAGGAACTTTCAGAA
	(R) GTCGGGAAGTTTTC CCACTCA	
BNP	(F) TGGGCAGAAGATAGACCGGA	CGGCGCAGTCAGTCGCTTGG
	(R) ACAACCTCAGCCC GTCACAG	
Ca α-actin	(F) GGGCCCTCCATTGTCCA	CGCAAGTGCTTCTGAGGCGGCTAC
	(R) GCACAATACTGTCGTCCTGAGTG	
IL-6	(F) CAGAATTGCCATTGCACAACTCTTTTCTCA	TGCATCATCGCTGTTCATACAA
	(R) ATATGTTCTCAGGG AGATCTTGGA	
α-MHC	(F) GCAGAAAATGCACGATGAGGA	TAACCTGTCCAGCAGAAAGAGCCTCGC
	(R) CATTCATATTTATTGTGGGATAGCAAC	
ß-MHC	(F) GCTACCCAACCCTAAGGATGC	TGTGAAGCCCTGAGACCTGGAGCC
	(R) TCTGCCTAAGGTGCTGTTTCAA	
OSP	(F) AATCGCCCCCACAGTCG	TGTCCCTGACGGCCGAGGTGA
	(R) CCTCAGTCCGTAAG CCAAGC	
PAI-1	(F) GCTGACCACAGCAGGGAAA	CCCGGCAGCAGATCCAAGATGCTAT
	(R) GTGCCCCTCTCACT GATATTGAA	
Sk α-actin	(F) TCCTCCGCCGTTGGCT	CATCGCCGCCACTGCAGCC
	(R)AATCTATGTACACGTCAAAAACAGGC	
18S	(F) TGGTTGCAAAGCTGAAACTTAAAG	CCTGGTGGTGCCCTTCCGTCA
	(R) AGTCAAATTAAGCC GCAGGC	

### Protein Synthesis

[4,5-^3^H] leucine incorporation was measured as previously described [20]. Briefly, cells were cultured in 24-well plates. When appropriate, the cells were transduced with recombinant adenoviruses. Subsequently the culture medium was supplemented with [4,5-^3^H] leucine (5 µCi/ml). After 24 h, cells were lysed and processed for measurement of incorporated [4,5-^3^H] leucine by liquid scintillation counter.

### Statistical analysis

The results are expressed as mean ± SEM. To determine the statistical difference between two groups, Student's *t*-test was used. For multiple comparisons, the results were analyzed with one-way analysis of variance (ANOVA) followed by a least significant difference (LSD) *post hoc* test. Differences at or above the 95% level were considered statistically significant.

## Results

### ATF3 gene expression is rapidly activated in response to ET-1 and mechanical stretch

**Figure 1:**
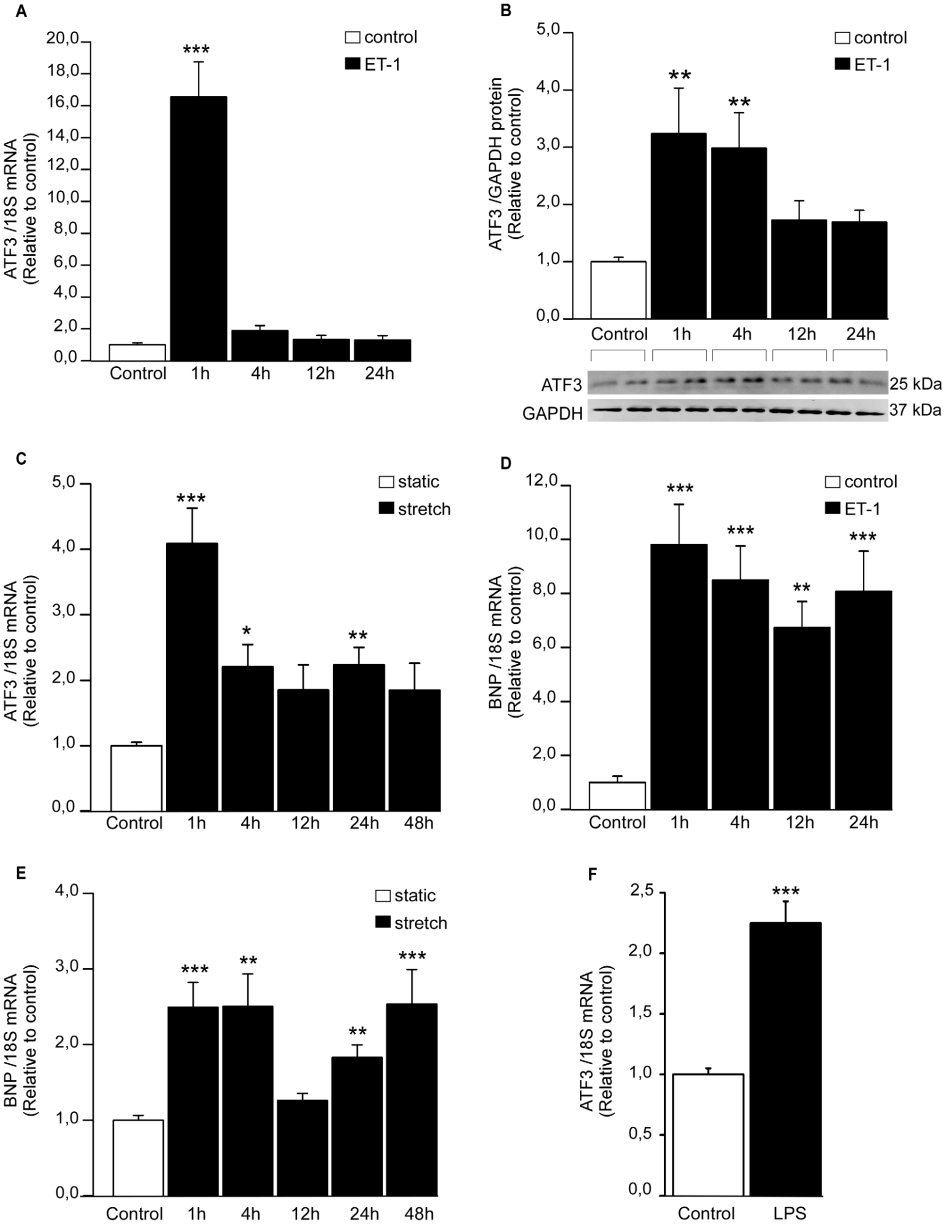
Hypertrophic stimuli induce ATF3 gene expression in cultured NRCM. Cardiomyocyte cell cultures were subjected to ET-1 (100 nM) stimulifor 1, 4, 12 and 24 hours (**A, B, D**), cyclic mechanical stretch for 1, 4, 12, 24 and 48 hours (**C, E**), or LPS (1 µg/ml) for 4 hours. ATF3 (**A, C, F**) and BNP (**D, E**) mRNA levels were normalized to 18S quantified from the same samples, and are presented relative to control cells. The results represent mean ± SEM (n = 8–18) from at least 3 independent experiments. **B,** ATF3 protein levels after ET-1 stimuli were determined by Western blotting and normalized to GAPDH loading control levels. Representative Western blots are shown, and bar graphs represent mean ± SEM (n = 7) from 3 independent experiments. *P<0.05; **P<0.01; ***P<0.001.

First, to directly examine the effect of hypertrophic stimuli on cardiomyocyte ATF3 expression, we treated NRCM with a panel of hypertrophic agonists *in vitro*. In agreement with previous studies showing that ET-1 activates ATF3 in NRCM [17], [18], ET-1 (100 nM) substantially increased ATF3 gene expression with maximal mRNA levels at 1 hour(16.6–fold; *P*<0.001), returning to almost basal levels within 4 hours (*Figure 1A*), while the increase in ATF3 protein levels was sustained up to 4 hours (*Figure 1B*). Furthermore, ATF3 mRNA levels were rapidly increased in response to cyclic mechanical stretching; the levels were highest (4.1–fold; *P*<0.001) at 1 h and remained significantly elevated also following 4- and 24-hours of stretch (*Figure 1C*). BNP mRNA levels, measured to validate hypertrophic response [22], were increased in response to both ET-1 (*Figure 1D*) and stretch (*Figure 1E*). Finally, when NRCM were treated with LPS (1 µg/ml), an increase in ATF3 mRNA levels was noted (*Figure 1F*).

### The effect of kinase inhibitors on ET-1-induced ATF3 activation

**Figure 2:**
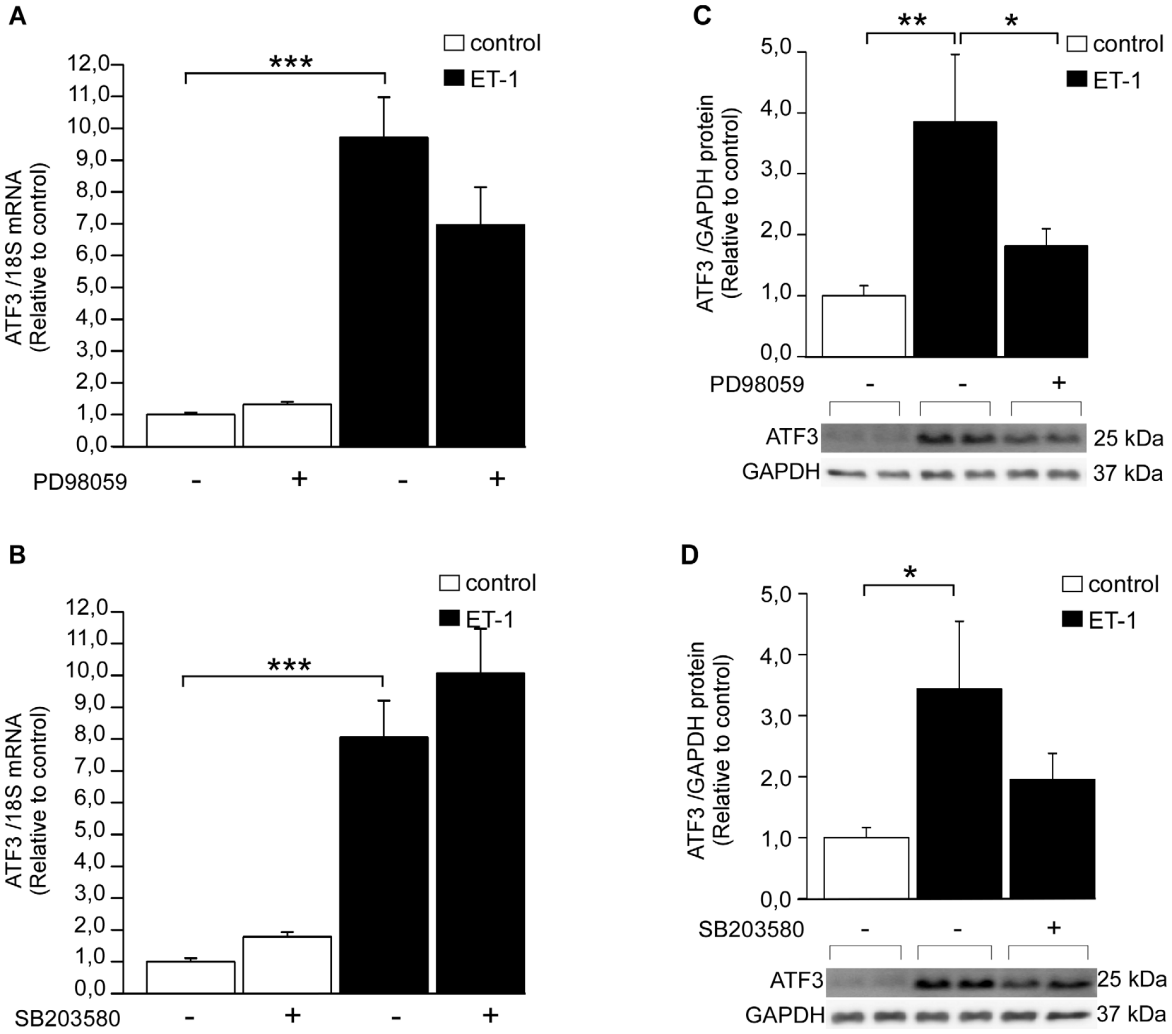
The effect of ERK and p38 MAPK kinase inhibition on ET-1–induced ATF3 expression. ERK inhibitor PD98059 (10 µM) (**A, C**) or p38 inhibitor SB203580 (10 µM) (**B, D**) were added to culture medium of neonatal rat cardiomyocyte cell cultures and 2 hours later ET-1 (100 nM) was added to medium for 1 hour. DMSO was used as a control. **A, B,** ATF3 mRNA levels were determined by qPCR and normalized to 18S quantified from the same samples. The mRNA levels are presented relative to non-stimulated DMSO control cells. The results represent mean ± SEM (n = 3–19) from at least 3 independent experiments. **C, D,** ATF3 and GAPDH protein levels were detected by Western blotting and representative Western blots are shown. ATF3 protein levels were normalized with GAPDH levels and are presented relative to non-stimulated DMSO control cells. Bar graphs represent mean ± SEM (n = 6–10) from at least 3 independent experiments. ** *P<*0.01; ***P<0.001.

**Figure 3:**
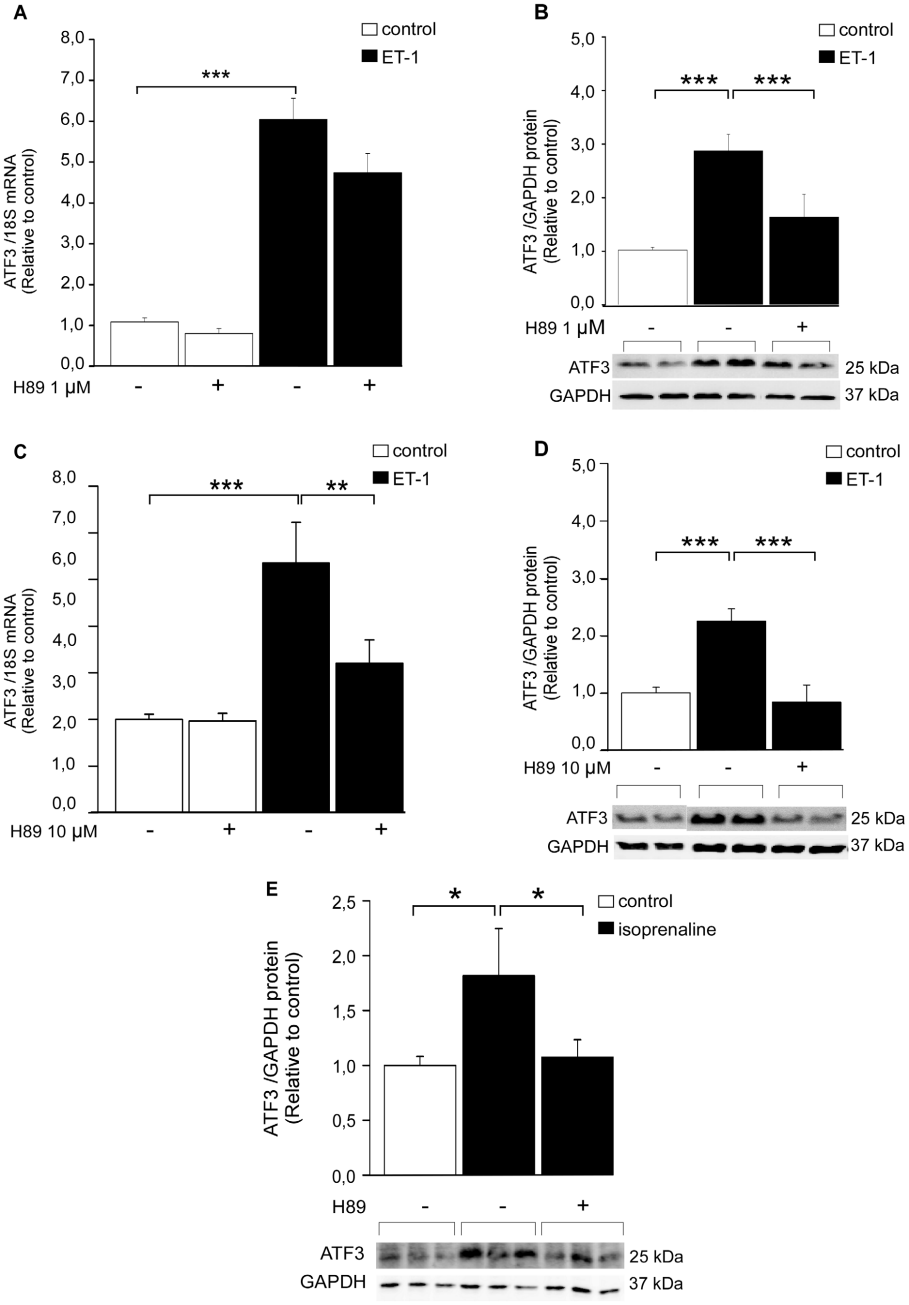
PKA inhibitor H89 inhibits ET-1- and isoprenaline-induced ATF3 increase in NRCM. Cultured cardiomyocytes were treated for 2 hours with PKA inhibitor H89 (1 µM **A, B** or 10 µM **C, D, E**) subsequently the cells were subjected to ET-1 (100 nM, 1 h) or isoprenaline (100 nM, 15 min) stimuli. **A, C,** ATF3 mRNA levels were determined by qPCR and normalized to 18S quantified from the same samples. The mRNA levels are presented relative to non-stimulated DMSO control cells. The protein levels of ATF3 and GAPDH loading control were detected by Western blotting (**B, D, E**). Representative Western blots are shown. Bar graphs represent ATF3 protein levels normalized with GAPDH levels and presented relative to control group. Mean ± SEM (n = 5–10) from 3 independent experiments is presented. *P<0.05; **P<0.01.

**Figure 4:**
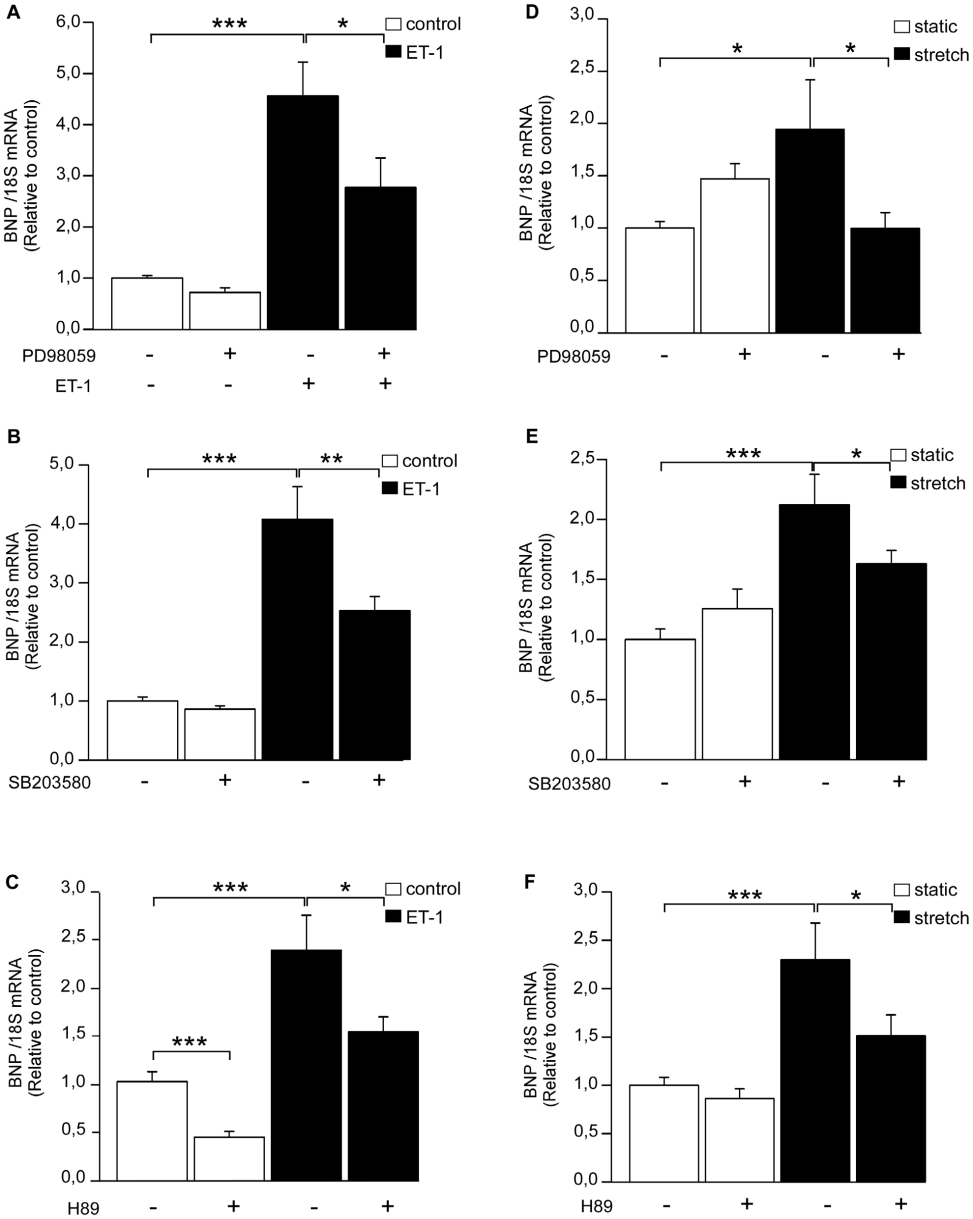
The effect of kinase inhibitors on ET-1 and mechanical stretch-induced BNP gene expression. ERK inhibitor PD98059 (10 µM) (**A, D**), p38 inhibitor SB203580 (10 µM) (**B, E**) or PKA inhibitor H89 (10 µM) (**C, F**) were added to culture medium of neonatal rat cardiomyocyte cell cultures 2 hours prior to 1 h ET-1 stimuli (100 nM; **A–C**) or cyclic mechanical stretching (**D–F**). DMSO was used as a control. The mRNA levels were normalized to 18S quantified from the same samples and the mRNA levels are presented relative to control cells. The results represent mean ± SEM (n =  8–18) from at least 3 independent experiments. *P<0.05; **P<0.01; ***P<0.001 compared to control.

Next, intracellular signaling pathways mediating ET-1 –induced ATF3 activation were studied in NRCM. ET-1 is a potent vasoconstrictor but also a pro-hypertrophic factor which is synthesized and secreted in response stretching of cardiomyocytes [26]. Similarly to mechanical stretch, ET-1 has been reported to induce activation of Raf-1 and MAPKs as well as protein kinases through the ET_A_ receptor, leading to higher protein synthesis and enlarged cell surface [26]. NRCM were exposed to kinase inhibitors SB203580 (10 µM), PD98059 (10 µM) or H89 (1 µM) [20], [23], [27]. Then, the cells were treated with 100 nM ET-1 for 1 hour. Neither PD98059 (*Figure 2A*), SB203580 (*Figure 2B*) nor H89 (*Figure 3A*) had significant effect on ET-1 –induced increase in ATF3 mRNA levels in NRCM. On the other hand, ET-1 –induced ATF3 protein expression was reduced by ERK inhibitor PD98059 (*Figure 2C*) and PKA inhibitor H89 (*Figure 3B*), indicating the involvement of posttranscriptional mechanisms, whereas a non-significant reduction was observed with p38 inhibitor SB203580 (*Figure 2D*). A higher dose of the PKA inhibitor H89 (10 µM, [27]) significantly decreased ET-1 –induced ATF3 mRNA expression (*Figure 3C*) and completely abolished increase in ATF3 protein levels (*P<*0.001; *Figure 3D*). Therefore, the PKA activator and β-receptor agonist isoprenaline [28] was applied to NRCM to study further the possible role of adenylyl cyclase-cAMP-PKA pathway in ATF3 regulation. The isoprenaline treatment significantly increased ATF3 protein expression and this increase was completely abolished by PKA inhibitor H89 (*Figure 3E*). Moreover, as reported previously [28], ET-1-induced BNP mRNA transcription was blunted by all three kinase inhibitors (*Figure 4A–C*).

### Stretch-induced ATF3 activation is attenuated by PKA pathway –inhibitor

**Figure 5:**
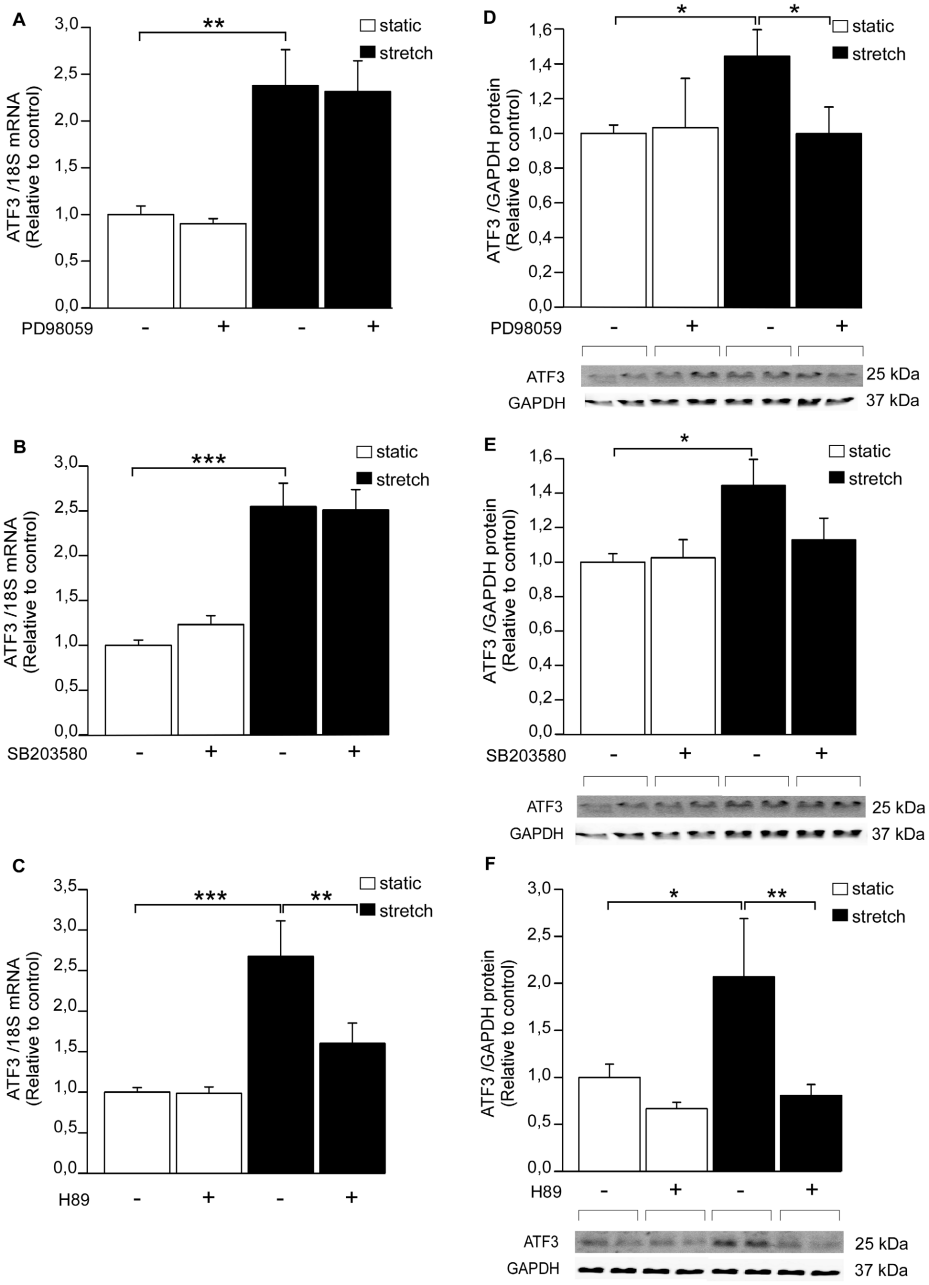
The effect of kinase inhibitors on mechanical stretch–induced increase in ATF3 expression. ERK inhibitor PD98059 (**A, D**), p38 inhibitor SB203580 (**B, E**) or PKA inhibitor H89 (**C, F**) were added at the concentration of 10 µM each. DMSO was used as a control. Approximately 2 hours after the insertion of inhibitors or DMSO, the cell cultures were subjected to cyclic mechanical stretching for 1 hour. ATF3 mRNA levels were determined by RT-qPCR and normalized to 18S quantified from the same samples (**A–C**). The mRNA levels are presented relative to non-stretched DMSO control cells. The results represent mean ± SEM (n = 2–19) from at least 3 independent experiments. The expression levels of ATF3 protein and GAPDH loading control were detected by Western blotting (**D–F**). Representative Western blots are shown. ATF3 protein levels were normalized with GAPDH levels and are presented relative to non-stretched DMSO control cells. Bar graphs represent mean ± SEM (n = 4–12) from at least 3 independent experiments. *P<0.05; **P<0.01; ***P<0.001.

Next, the effect of kinase inhibitors on mechanical stretch-induced ATF3 expression was studied in NRCM. Neither ERK inhibitor PD98059 (*Figure 5A*) nor p38 MAPK inhibitor SB203580 (*Figure 5B*) diminished stretch-activated increase in ATF3 mRNA levels at one hour. Instead, ERK inhibition abrogated the stretch-induced increase of ATF3 protein levels (*Figure 5D*) and p38 inhibitor resulted in a similar trend (*Figure 5E*). Moreover, H89 attenuated one-hour mechanical stretch-induced increase in ATF3 mRNA levels (*Figure 5C*) and completely abolished stretch-induced increase in ATF3 protein expression (*Figure 5F*). All kinase inhibitors significantly decreased the mechanical stretch-induced BNP gene expression (*Figure 4D–F*), indicating that the time point and dose of the inhibitors used in these experiments were sufficient to reduce stretch-induced gene expression in cardiomyocyte cultures.

### ATF3 activation is distinctly regulated by the two p38 MAPK isoforms

**Figure 6:**
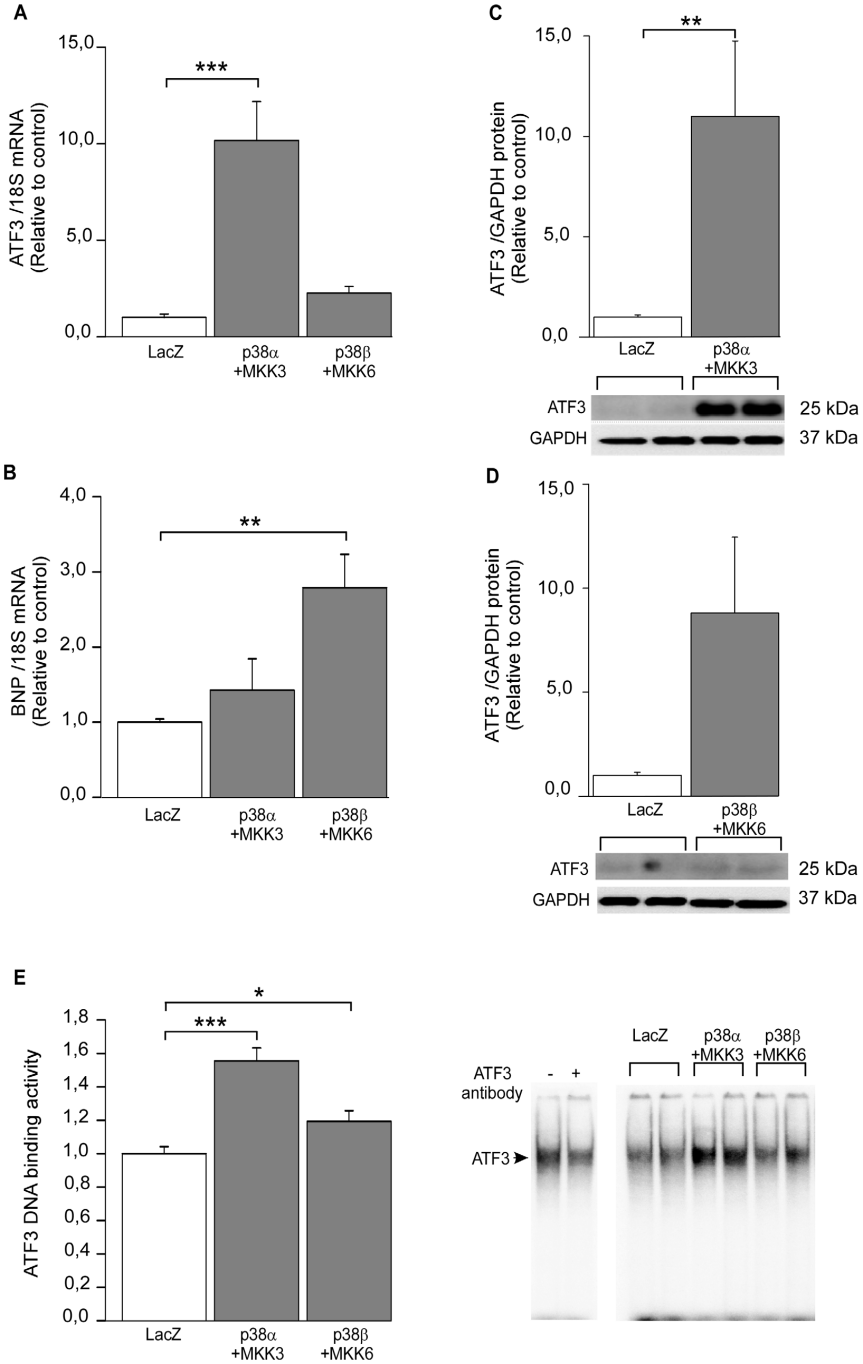
p38α MAPK regulates ATF3 activity. Cultured cardiomyocytes were transduced with recombinant adenoviruses WT p38α, WT p38β, MKK3b and/or MKK6b for 24 hours at the virus amount of 4 MOI (2+2 MOI in combinations). RT-qPCR with cDNA derived from mRNA of neonatal rat cardiomyocyte cell cultures transduced with recombinant adenovirus combinations WT p38α+MKK3b, WT p38β+MKK6b or control virus LacZ (**A–B**). ATF3 (**A**) or BNP (**B**) mRNA levels were normalized to 18S quantified from the same samples and the mRNA levels are presented relative to LacZ control cells. The results represent mean ± SEM (n = 6–8) from 4 independent experiments. Western blot analysis of cell lysate derived from NRCM cultures transduced with WT p38α and MKK3b (**C**) or WT p38β and MKK6b (**D**) recombinant adenoviruses. ATF3 and GAPDH protein levels were detected by Western blotting and representative Western blots are shown. ATF3 protein levels were normalized with GAPDH levels and are presented relative to LacZ control. Bar graphs represent mean ± SEM (n = 6) from 3 independent experiments. EMSA of nuclear protein from adenovirus–transduced cultured cardiomyocytes (**E**). ATF3 antibody (2 µl) causes supershift reaction (representative blot is shown) and ATF3 binding activity in response to WT p38α+MKK3b and WT p38β+MKK6b is presented as bar graphs (mean ± SEM, n = 12–13 from 3 independent experiments) and representative blot. * *P<*0.05; **P<0.01; *** *P<*0.001.

Since we have previously observed that ATF3 is a target gene of p38α MAPK [12] [29], we next examined possible differences between the two main cardiac p38 isoforms, p38α and p38β, in regulation of ATF3 activation. In view of previous observations [20], [30], cardiomyocyte cell cultures were transduced with recombinant adenoviruses coding WT p38α and WT p38β along with their constitutively active upstream kinase MKK3b and MKK6b, respectively. ATF3 mRNA levels were significantly increased by p38α+MKK3b overexpression, while p38β+MKK6b had no significant effect on ATF3 gene expression (*Figure 6A*). BNP mRNA levels were in turn significantly enhanced by p38β+MKK6b and not by p38α+MKK3b (*Figure 6B*), as previously shown [20]. ATF3 protein expression was also increased in response to p38α +MKK3b (*Figure 6C*). The p38β isoform markedly increased ATF3 protein expression although it was not statistically significant (*Figure 6D*). Finally, to investigate whether the higher ATF3 protein levels are associated to higher amount of protein capable of binding a consensus ATF3 target binding site, we performed an EMSA assay. As shown in *Figure 6E*, ATF3 DNA binding activity was promoted in response to p38α+MKK3b and to lesser extent with p38β+MKK6b.

### Role of ATF3 in the regulation of cardiomyocyte hypertrophy

**Figure 7:**
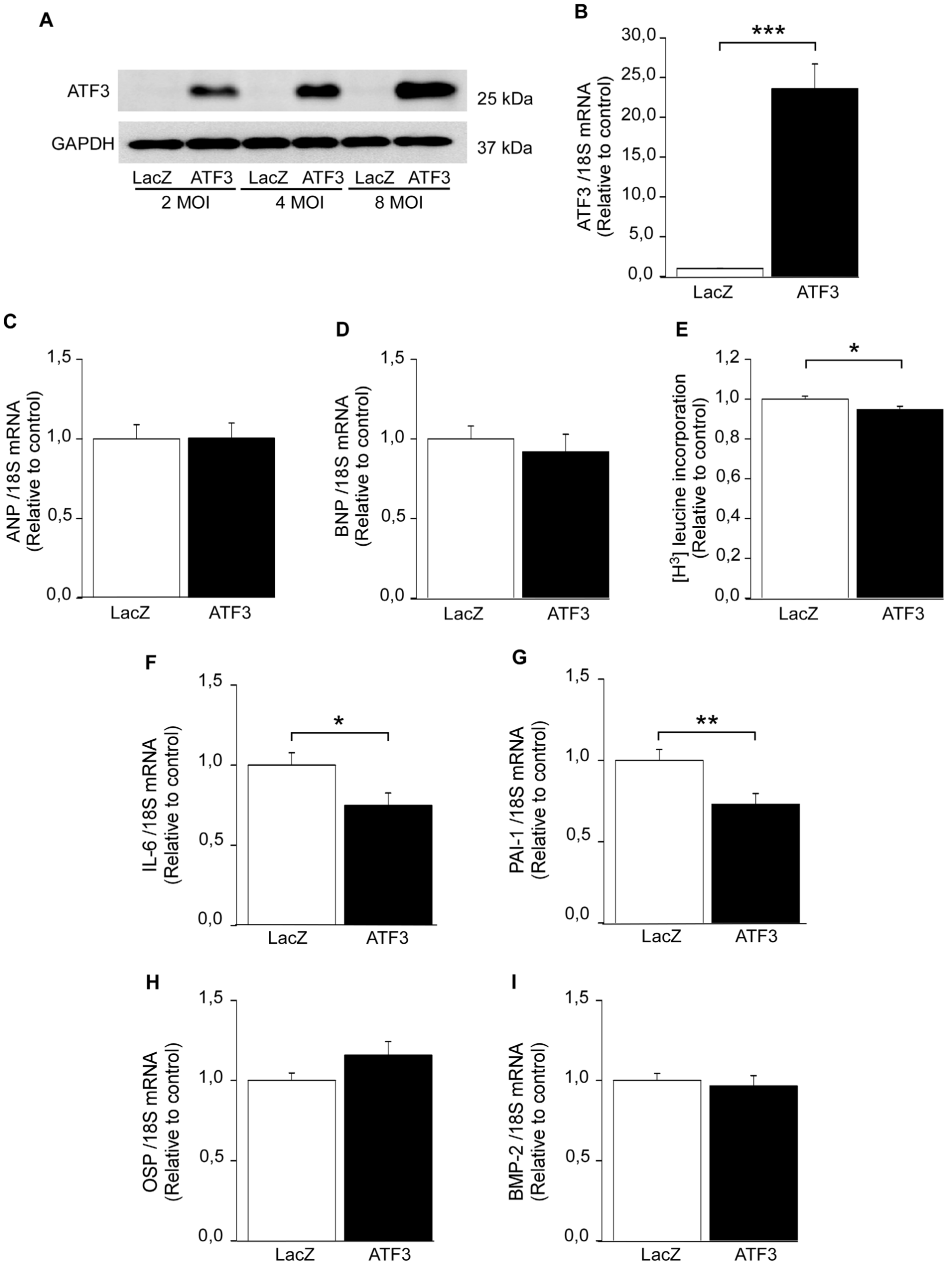
The effect of ATF3 overexpression *in vitro* on hypertrophy and inflammatory markers. Representative Western blot analysis of cell lysate derived from cultured NRCM transduced with recombinant adenoviruses overexpressing ATF3 or control virus LacZ at the virus amounts of 2, 4, and 8 MOI (**A**). The expression level of ATF3 (*upper panel*) and GAPDH loading control (*lower panel*) from one representative Western blot (performed in triplicates) is shown. RT-qPCR analysis with cDNA derived from mRNA of NRCM cultures transduced with recombinant adenovirus ATF3 or control virus LacZ (**B–D, F–G**). ATF3 (**B**), ANP (**C**), BNP (**D**), IL-6 (**F**), PAI-1(**G**), OSP (**H**), and BMP-2 (**I**) mRNA levels are normalized to 18S quantified from the same samples and presented relative to LacZ control cells. The results represent mean ± SEM (n = 27–32) from 3- 4 independent experiments. [^3^H]leucine (5 µCi/ml) was inserted to culture medium of NRCM transduced with recombinant adenovirus ATF3 or control virus LacZ at the concentration of 4 MOI, and incorporated [^3^H]leucine was detected by liquid scintillation counter (**E**). The results represent mean ± SEM (n = 32) from 3 independent experiments. *P<0.05; ***P<0.001.

To investigate the role of ATF3 in hypertrophic process, we used gain-of-function approach and overexpressed ATF3 in NRCM by adenoviral transfection. The adenovirus- mediated ATF3 gene delivery markedly increased ATF3 protein levels at the virus amounts of 2, 4 and 8 MOI (*Figure 7A*) and ATF3 mRNA levels were also significantly increased in response to adenoviral ATF3 overexpression (*Figure 7B*). Of note, the molecular weight of endogenous ATF3 (for example, see *Figure 1B*) was similar to ATF3 produced by adenovirus-mediated gene delivery. Interestingly, the expression of natriuretic peptides ANP (*Figure 7C*) and BNP (*Figure 7D*) were not altered by the overexpression of ATF3. Furthermore, another hallmark feature of cardiomyocyte hypertrophy, the rate of protein synthesis [31], was slightly but significantly decreased (−5.3%, *P<*0.05) in response to ATF3 overexpression, as measured by radioactively labeled leucine ([^3^H] leucine) incorporation assay (*Figure 7E*).

**Figure 8:**
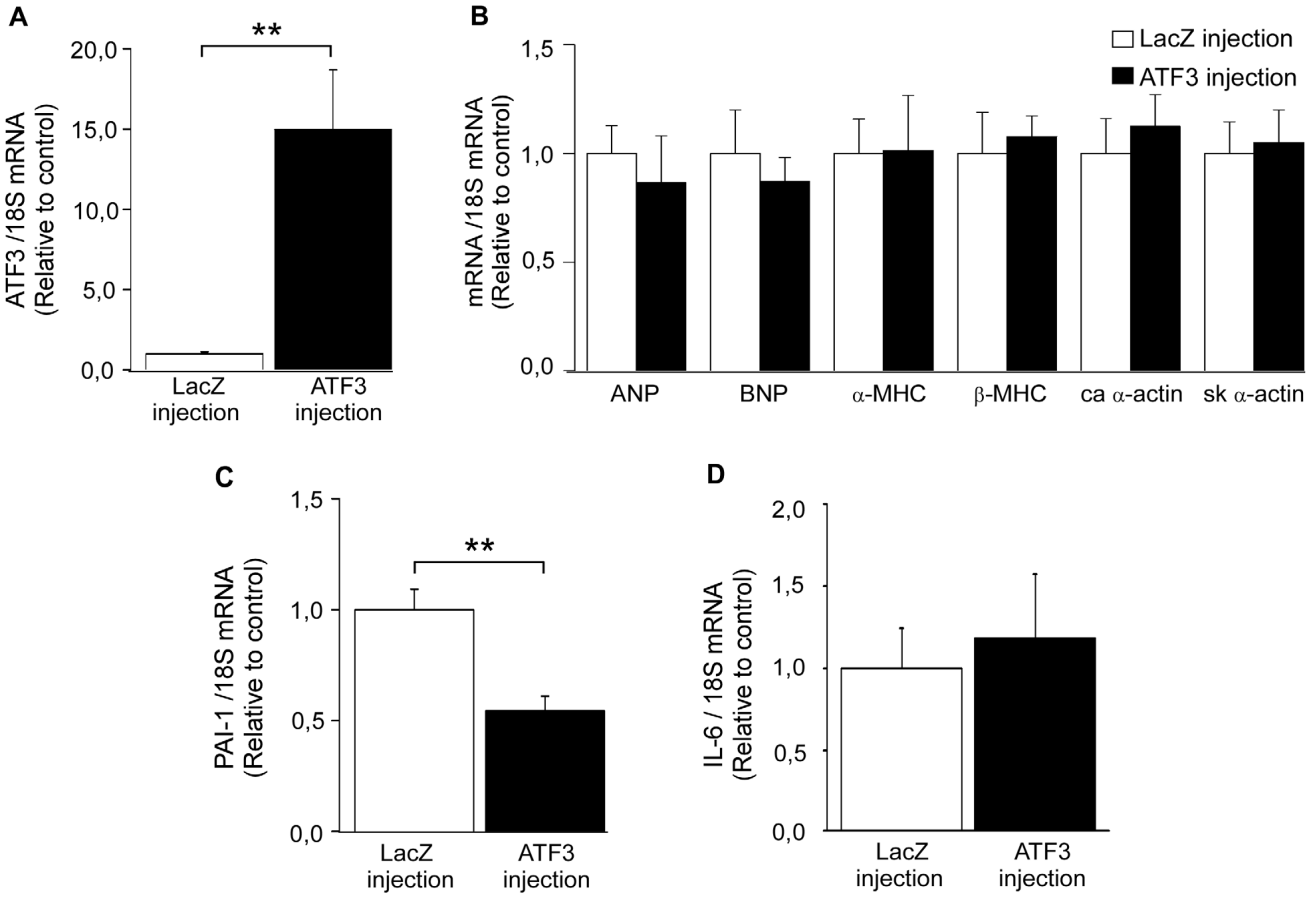
*In vivo*ATF3 overexpression downregulates the gene expression of PAI-1. Three days following the adenoviral injection of ATF3 or LacZ (1×10^9^ pfu) into the left ventricular wall of adult rats, the animals were sacrificed and mRNA was extracted and cDNA synthesized for RT-qPCR analysis. ATF3 (**A**), ANP, BNP, α- and ß-MHC, cardiac and skeletal α-actins (**B**), PAI-1 (**C**) and IL-6 (**D**) mRNA levels were normalized to 18S quantified from the same samples and presented relative to LacZ-injected animals. The results are mean ± SEM (n = 4–5). *P<0.05; **P<0.01.

To determine whether ATF3 has a role in hypertrophic response *in vivo*, ATF3 was overexpressed by using adenovirus-mediated gene delivery in normal adult rat heart for 3 days. This time point was chosen because our previous time-course studies indicated that the maximal up-regulation of target gene expression is observed at day 3 after gene transfer [12]. ATF3 mRNA levels were increased 15.0-fold in response to adenoviral ATF3 overexpression at three days following injections (*Figure 8A*). On the other hand, ATF3 adenoviral gene transfer *in vivo* had no effect on the gene expression of the several cardiac hypertrophy markers, including ANP, BNP, α-MHC, and ß-MHC, and skeletal and cardiac α-actins (*Figure 8B*) or the structure and function of the heart, as analyzed by echocardiography (*Table 3*).

**Table 3:** Echocardiography, and body and left ventricular weight data of ATF3 adenovirus-mediated gene delivery in normal adult rat heart compared to LacZ control.

Variable	LacZ	ATF3	*P-value*
IVS(diastole, mm)	2.0±0.1	1.8±0.1	*0.151*
LV (diastole, mm)	7.7±0.3	7.4±0.1	*0.524*
LVPW (diastole, mm)	1.6±0.1	1.8±0.1	*0.561*
Fractional shortening (%)	36.6±0.7	34.1±0.1	*0.268*
Ejection fraction (%)	72.0±0.9	68.6±0.1	*0.239*
LV IVRT	22.5±1.4	25.8±0.1	*0.083*
HR	286±9	270±0.1	*0.213*
LV mass (mg)	1027±48	970±0.1	*0.588*
Body weight T0 (g)	274±7	256±0.1	*0.125*
Body weight T3 (g)	281±9	259±0.1	*0.084*
BW T3-T0 (g)	7±5	3±0.1	*0.511*
LV mass/BW (mg/g)	3.7±0.2	3.8±0.4	*0.913*

Adenoviral gene construct expressing ATF3 and LacZ were injected onto the LV free wall and echocardiographic measurements were performed at 3 days after gene transfer. The values are expressed as mean ± SEM (n = 5–6/group).

*P-values* are the result of ATF3 vs. LacZ Student's t-test.

IVS, interventricular septum; LV, left ventricle; LVPW, left ventricular posterior wall; IVRT, isovolumetric relaxation time; HR, heart rate; BW, body weight.

### ATF3 overexpression down-regulates inflammation–related genes

ATF3 has been proposed to be involved in the cardiac inflammatory response [16], [17]. Since we observed only a modest effect on the hypertrophic response, the possible role of ATF3 as a regulator of inflammation was explored by investigating the effect of increased ATF3 on the expression of IL-6, PAI-1, OSP and BMP-2 genes. All these genes represent important mediators in cardiac pathology, mainly in inflammation and remodeling process [32]–[34]. In agreement with previous studies [16], [17], ATF3 overexpression in cultured NRCM significantly reduced IL-6 mRNA levels (*Figure 7F*). Also PAI-1 mRNA levels were decreased (*Figure 7G*), while OSP (*Figure 7H*) and BMP-2 mRNA (*Figure 7I*) levels remained unchanged. Importantly, overexpression of ATF3 *in vivo* resulted in a significant decrease in PAI-1 mRNA levels (*Figure 8C*), although it had no effect on IL-6 mRNA levels (*Figure 8D*). This may be due to different experimental conditions (neonatal versus adult cells). It should be noted that *in vitro* changes occurring to a single cell type were studied, while in vivo cardiomyocytes co-exist with other cell populations.

### Overexpression of ATF3 results in increase in Nkx-2.5 and NF-κB DNA binding activities

**Figure 9:**
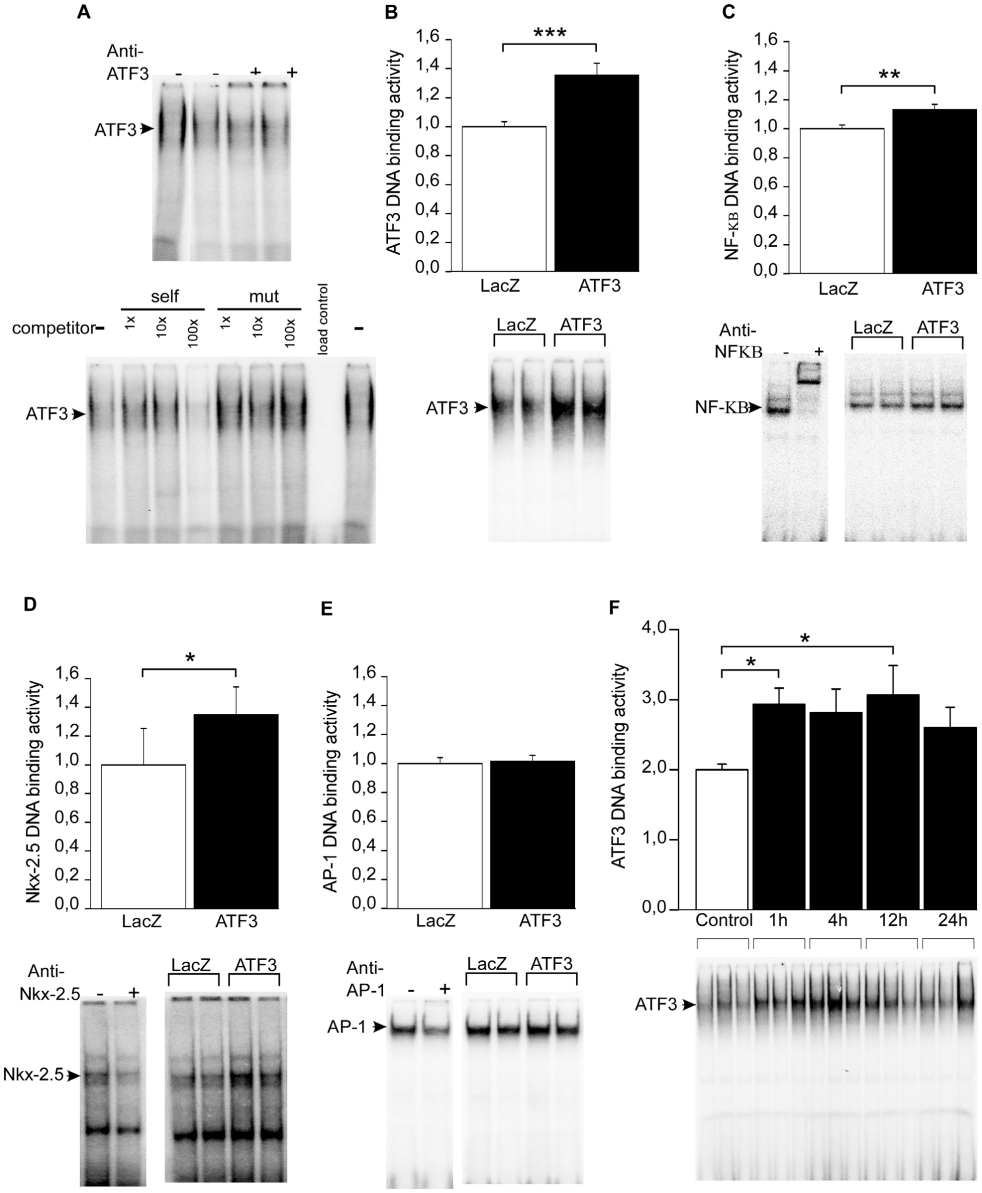
ATF3 overexpression induces the binding activity of NF-κB and Nkx-2.5. Representative EMSA blot of nuclear protein from cultured NRCM (**A**). ATF3 antibody (2 µl) caused supershift reaction (*upper panel*) and non-radiolabeled ATF3 probe (self) competes with radiolabeled ATF3 for binding to the probe, whereas mutated non-radiolabeled ATF3 probe (mut) had no effect (*lower panel*). EMSA of nuclear protein from ATF3 or LacZ adenovirus transduced (for 24 hours at the virus amount of 4 MOI) cultured cardiomyocytes (**B–E**). ATF3 (**B**), NF-κB (**C**), Nkx-2.5 (**D**) and AP-1 (**E**) binding activities are presented as bar graphs (mean ± SEM, n = 9–15 from at least 3 independent experiments) and representative blots. Also NF-κB (**C**), Nkx-2.5 (**D**) and AP-1 (**E**) antibodies (2 µl) cause supershift reactions (representative blots are shown). ET-1 stimulates ATF3 binding activity in cardiomyocytes (**F**). EMSA of nuclear protein from cultured cardiomyocytes treated with ET-1 (100 nM) for 1, 4, 12 and 24 hours. ATF3 binding activity is presented as bar graphs (mean ± SEM, n = 8 from 3 independent experiments) and representative blot. *P<0.05; **P<0.01; ***P<0.001.

Since our results indicate that ATF3 modulates rather inflammatory than hypertrophic factors, we investigated the effects of ATF3 overexpression on DNA binding activity of transcription factors known to be involved in the regulation of inflammatory genes such as NF-κB, Nkx-2.5 and AP-1 transcription factors. The cardiomyocyte nuclear extracts exhibited specific binding activity on ATF3 binding site; the formation of complexes with the ATF3 probe was dose-dependently inhibited by the non-radiolabeled ATF3 dsDNA oligonucleotides with intact binding site, but not by the oligonucleotides with mutated binding site, and supershift analysis showed antibody-induced supershift of the ATF3 complex (*Figure 9A*). ATF3 binding activity was elevated 35.6% (*P<*0.001) in response to ATF3 overexpression (*Figure 9B*) further indicating that the ATF3 produced by adenovirus-mediated gene delivery was functional. Importantly, the DNA binding activities of NF-κB (*Figure 9C*) and Nkx-2.5 (*Figure 9D*) were significantly increased in response to ATF3 overexpression, whereas AP-1 binding activity was not changed (*Figure 9E*). As a control experiment for increased ATF3 DNA binding, we studied the changes in ATF3 binding activity in response to ET-1 stimulation. ET-1-insertion into culture medium augmented ATF3 binding activity by 31.8% at 1 h and this increase was sustained at 4 h, 12 h and 24 h time-points (*Figure 9F*).

## Discussion

Myocardial hypertrophy can be induced by pressure or volume overload, and also by a number of neurohumoral factors, including ET-1, angiotensin II, noradrenaline [31], and isoprenaline [13]. *In vitro* mechanical stretch provides a reliable model whose effects on cardiomyocytes resembles those of cardiac pressure overload-induced cardiac hypertrophy *in vivo*. ATF3 is an immediate early gene (IEG), which is typically induced by growth factors or various stress signals [35] suggesting that it may be a key regulator in cellular stress responses. It is assumed that rapid and transient induction of IEGs such as ATF3, *c-fos,c-jun* and EGR-1 regulate the expression of other hypertrophic genes [17], [36]. This study demonstrates that ATF3 is substantially activated in response to ET-1 treatment, in agreement with previous studies [16], [17]. Furthermore, ET-1 stimulation significantly increased ATF3 DNA binding activity, detected by EMSA. Our results also for first time demonstrate that ATF3 is potently and rapidly (within 1 hour) activated by direct mechanical stretch of cardiomyocytes.

The involvement of JNK pathway in the regulation of ATF3 activation in various cells is well established [37]–[39], whereas the role of ERK is more controversial; it has been shown to function as a positive regulator of ATF3 in human colorectal cancer cells [40] and negative regulator of TNFα (tumor necrosis factor α) - mediated induction of ATF3 in vascular endothelial cells [38]. Moreover, the inhibition of ERK pathway by PD98059 had no effect on anisomycin-induced ATF3 activation and overexpression of ERK or upstream activator MKK1 did not increase the ATF3 transcription in HeLa cells [41]. Here we show that the inhibition of ERK pathway with MKK1 inhibitor PD98059 attenuated the ET-1– and stretch-induced ATF3 protein expression but not ATF3 mRNA transcription. These data indicate that ERK regulates ATF3 transcription through posttranscriptional modifications. The inhibition of p38 MAPK by SB203580 had only a modest and statistically non-significant effect on ATF3 protein levels and no effect on ATF3 mRNA level, thus implicating that inhibiting p38 does not significantly impair ET-1– or stretch- induced ATF3 activation.

Our results further demonstrate that the PKA inhibitor H89 is able to prevent the ET-1- and mechanical stretch– induced ATF3 gene and protein expression in NRCM. Interestingly, a study showed that ATF3 overexpression in cardiomyocytes diminished phospholamban (PLB) promoter activation [42]. PLB in turn, is a target of PKA-mediated phosphorylation leading to enhanced systolic Ca^2+^ concentrations improving cardiac contractility [28]. Although the selectivity of H89 as PKA inhibitor has been argued [43], [44], H89 is still widely used as PKA inhibitor [27], [45]. Our results show that H89 prevents ATF3 mRNA expression also in the presence of isoprenaline, a more specific PKA activator known to activate ATF3 [13]. Thus, it can be hypothesized that ATF3 might play a role in PKA-mediated regulation of cardiac muscle contractility. Consistent with this, ATF3 is also induced by adenylyl cyclase VI overexpression in cardiomyocytes [42].

Even though we observed a very modest effect of p38 MAPK route blockade on ATF3, our previous DNA microarray study demonstrated that p38α MAPK overexpression with MKK3b induced ATF3 mRNA expression 7.7-fold [12]. Therefore, we characterized in detail the possible differences between the two main p38 isoforms in the heart, p38α and p38β, in the regulation of ATF3. Notably, upstream kinase MKK6, which is 80% homologous to MKK3, can activate all four p38 isoforms (p38α, p38β, p38γ, and p38δ), whereas MKK3 preferentially activates only p38α, p38γ, and p38δ [30]. We have previously reported significant differences in the downstream targets of p38α and p38β [20]. Here we show that p38α isoform overexpression in combination with MKK3 increased ATF3 mRNA and protein expression as well as ATF3 DNA binding activity. Taken together, the regulation of ATF3 by p38 pathways is isoform specific, since only p38α overexpression was able to activate ATF3.

Although ATF3 gene expression has been consistently reported to be induced in ischemic and ischemia/reperfusion-treated hearts *in vivo* and *in vitro* [10], [11], [39], the role of ATF3 in cardiovascular pathology is unclear. Transgenic mice expressing ATF3 under the control of the α-myosin heavy chain promoter exhibited both atrial enlargement and ventricular hypertrophy as well as myocyte degeneration, extensive fibrosis of the heart wall, conduction abnormalities and contractile dysfunction [18], [19], indicating that ATF3 is detrimental stress-inducible gene. In contrast, Nobori *et al.* demonstrated cardioprotective effects of the acute induction of ATF3 [46]. In addition, more recent studies using loss-of-function approaches resulted in impaired hypertrophic response *in vitro* [16], [17] while *in vivo* it revealed promotion of cardiac hypertrophy, dysfunction and fibrosis [15]. Here we show that the overexpression of ATF3 did not affect the gene expression of cardiac hypertrophy markers such as the natriuretic peptides ANP and BNP, α- and β-MHC, or cardiac and skeletal α-actin isoforms. Both ANP and BNP are rapidly induced during mechanical loading of cardiomyocytes both *in vitro* and *in vivo* and highly expressed in hypertrophied left ventricles [3], [47]. Moreover, the rate of protein synthesis, another important hallmark of pathological cardiac hypertrophy, was not increased but even slightly decreased in response to ATF3 overexpression. Thus, although ATF3 was induced by hypertrophic stimuli, the ATF3 activation was not coupled with the central elements of pathological left ventricular hypertrophy – induction of hypertrophic genes and increased rate of protein synthesis – suggesting that the role of ATF3 in stressed cardiac myocytes might in fact be beneficial [15], [17].

NF-κB repression has been shown to rescue cardiac function and improve survival during cardiac inflammation through decreased apoptosis [8]. NF-κB is also a central regulator of cardiac hypertrophy and it controls the expression of IEGs as well as stress-responsive genes in many cell types [8]. For example, it has been shown that NF-κB mediates both isoprenaline- and angiotensin II–induced cardiac hypertrophy and inflammation *in vivo* [48]. Nkx-2.5 transcription factor, in turn, is a critical regulator of cardiac development and suggested to participate in cardiac hypertrophic response through its known ability to interact with other cardiac transcription factors such as GATA4 and serum response factor (SRF) [8]. Previously it has been shown that PE and isoprenaline–induced hypertrophic response is partly mediated through Nkx-2.5 [49]. Accumulated evidence also indicates that Nkx-2.5 functions as a survival factor for cardiomyocytes [8]. Using EMSA, we demonstrated in the current study that ATF3 overexpression increased DNA binding activity of NF-κB and Nkx-2.5, while the binding activity of AP-1, transcription factor mainly related to pathological cardiac hypertrophy [7], [50], remained unchanged. Taken together, these data support the hypothesis that ATF3 is involved in an adaptive hypertrophic response through activation of NF-κB andNkx-2.5.

In addition to hypertrophy, myocardial fibrosis plays a key role in development of heart failure [1], [2]. Fibrosis involves the accumulation of collagens I and III in the myocardium and the activation of numerous pro-fibrotic molecules such IL-6 and PAI-1, as well as vasoactive substances angiotensin II and ET-1 [16], [17]. It has been previously demonstrated that ATF3 is a potential repressor of ET-1–induced IL-6 activation, and an ATF3 consensus sequence is present in rat IL-6 promoter [16], [17], [51]. IL-6 is an inflammation-associated gene elevated in pathological hypertrophy, and the elevation of IL-6 has been linked to the development of heart failure [52]. PAI-1, in turn, is a member of serine protease inhibitor superfamily, shown to play a key role in the regulation of proteolytic degradation of the extracellular matrix during cardiac remodelling process [53]. IL-6 has been shown to be up-regulated by p38α overexpression *in vivo* [12] and PAI-1 by angiotensin II infusion *in vivo* [53]. In contrast, ATF3 knockdown in ET-1 stimulated cardiomyocytes had no effect on increase in expression of IL-6, suggesting that ATF3 regulates IL-6 through interaction with NF-κB rather than ERK½, which is up-regulated by ET-1 stimuli [17]. In adult mice, IL-6 and other inflammatory markers were increased due to ATF3 expression [18]. We observed here that IL-6 gene expression is significantly decreased *in vitro* in response to ATF3 overexpression, as reported previously [12]. In addition, ATF3 overexpression significantly diminished PAI-1 gene expression both *in vivo* and *in vitro*, suggesting an anti-fibrotic function for ATF3. On the other hand, the levels of two other markers of cardiac pathology, BMP-2 and osteopontin OSP [33], [54] remained unchanged in response to ATF3 activation. Collectively, these studies are consistent with the role of ATF3 as hypertrophic stimuli -inducible transcriptional repressor of gene expression [10], [55].

In conclusion, our present findings demonstrate that ATF3 transcription and DNA binding activity are up-regulated by hypertrophic stimuli *in vitro*. A number of signalling pathways are activated in cardiomyocytes after stress stimuli, and our results demonstrate ATF3 activation is partly mediated through ERK, PKA and p38α but not p38β. Finally, our observations support a cardioprotective role for ATF3 through induction of survival factor Nkx-2.5, and through attenuation of pro-fibrotic and pro-inflammatory proteins IL-6 and PAI-1. Our results, in combination with previous studies, indicate that ATF3 is involved in an adequate early response to stress-stimuli in heart and suggest that dysregulation of ATF3 signal transduction might contribute to maladaptive response and, therefore, to the development of heart failure.

## Limitations

One limitation in evaluating the effects of hypertrophic stimuli in cardiomyocytes was that we did not explore the possibility of changes in the subcellular localization of ATF3, possibly explaining differences between ATF3 mRNA expression and protein levels. Furthermore, cardiomyocyte-specificity of the adenovirus-mediated gene delivery *in vivo* was not assessed, and thus we cannot rule out the possibility of the effects of other cell populations. In addition, to reveal the long-term structural and functional effects of ATF-3 overexpression, the experiments with long term follow-up are needed.
